# A balancing act: Navigating the advantages and challenges of pioneering mycetoma treatment in Sudan—A landmark trial by the Mycetoma Research Center

**DOI:** 10.1371/journal.pntd.0013000

**Published:** 2025-04-21

**Authors:** Ahmed Hassan Fahal, Eiman Siddig Ahmed, Ali Awadallah Saeed, Lamis Ahmed Fahal, Samira M. E. Hussein, Kyoko Nakano, Katsura Hata, Fabiana Alves, Borna A. Nyaoke

**Affiliations:** 1 The Mycetoma Research Center, University of Khartoum, Khartoum, Sudan; 2 Eisai Co., Ltd., Tokyo, Japan; 3 Drugs for Neglected Diseases Initiative, Geneva, Switzerland; 4 Drugs for Neglected Diseases Initiative, Nairobi, Kenya; Albert Einstein College of Medicine, UNITED STATES OF AMERICA

## Abstract

The global burden of mycetoma, a debilitating, neglected tropical disease, is unknown, and patients struggle to complete treatment due to limited accessibility and affordability of medications. This communication highlights a landmark clinical trial conducted by the Mycetoma Research Center (MRC) at the University of Khartoum, Sudan, in partnership with the Drugs for Neglected Diseases initiative (DNDi) and Eisai Co., Ltd. (Eisai). Published in The Lancet Infectious Diseases, this clinical trial marks a significant advancement in mycetoma research and treatment. As the first randomised clinical trial assessing a new mycetoma treatment, it compared fosravuconazole with the current standard of care, itraconazole. While the trial found no dose of fosravuconazole to be superior to itraconazole, it did reveal that fosravuconazole presented no new safety concerns. Moreover, its lower pill burden, reduced risk of drug–drug interactions, and the fact that it can be taken without food make it a more feasible alternative to the relatively expensive and less accessible itraconazole for treating eumycetoma. This clinical trial, conducted in a difficult socio-political situation in Sudan, was only made possible by the exceptional efforts of the MRC. This groundbreaking study not only advances treatment options for mycetoma but also enhances research capacity in an endemic region, paving the way for future investigations into neglected tropical diseases.

## Background

Mycetoma is a WHO-recognised neglected tropical disease that chronically affects the subcutaneous tissues [1]. It occurs in the mycetoma belt, stretching between latitudes of 1.5° South and 30° North. Mycetoma is of two types: actinomycetoma, caused by certain bacteria, and eumycetoma, caused by true fungi [2]. The common causative organisms are *Nocardia brasiliensis* and *Madurella mycetoma*, respectively. The treatment depends on the underlying causative organism, site, and severity of the disease, with actinomycetoma being more responsive to medical treatment, with a cure rate of up to 90%. On the other hand, eumycetoma treatment is often challenging and suboptimal, necessitating surgical intervention alongside medical therapy [3]. The Mycetoma Research Center (MRC) established guidelines recommending the use of co-amoxiclav in combination with co-trimoxazole as a first-line treatment for actinomycetoma. While itraconazole is used as the first line for the treatment of eumycetoma. Folic acid is used in both types of mycetoma [4,5].

## The clinical trial

This first-ever randomised, double-blind clinical trial for a new mycetoma treatment marks a critical moment in the global fight against this debilitating, neglected tropical disease, which has long suffered from limited treatment options and a lack of research on the development of new therapies [6]. This groundbreaking trial, conducted by the MRC at the University of Khartoum, Sudan, and the Drugs for Neglected Diseases initiative (DNDi), in collaboration with Eisai, represents a significant milestone in mycetoma research and treatment.

Mycetoma, often dubbed “the forgotten disease,” has plagued tropical and subtropical regions for centuries [7–9]. This chronic fungal or bacterial infection, usually acquired through traumatic inoculation of microorganisms from the environment, penetrates deep beneath the skin into the subcutaneous tissue, affecting the bones and surrounding tissues [2,10].

The consequences of mycetoma are devastating, leading to grotesque swelling, disfigurement, and often loss of function in the affected limbs [11–14]. Beyond the physical suffering, it also carries profound social stigma, resulting in isolation, discrimination, and economic suffering for patients and their families [11]. This is due to the affected patients’ low health education and socioeconomic status and, limited healthcare facilities in remote communities, and hence their late presentation for treatment.

For far too long, mycetoma has languished in the shadows of global health priorities, receiving scant attention and research funding [7,15]. Patients have endured prolonged and often ineffective treatment regimens, relying on outdated medications with severe side effects (e.g. ketoconazole), multiple, often disfiguring surgeries, and ultimately amputation of the affected limb [3,16]. In many endemic regions, access to even these limited treatment options remains a distant dream for those living in poverty and in remote communities [17,18].

The MRC, recognising the dire need for safe and effective mycetoma treatments and drawing upon its deep well of expertise, partnered with the DNDi, an NGO committed to delivering new treatments for neglected diseases. Together, they embarked on a mission to evaluate the efficacy and safety of fosravuconazole, a promising new antifungal agent discovered by Eisai, with a favourable safety profile and convenient once-weekly dosing regimen [6].

This ambitious undertaking, a randomised, double-blind, phase 2, proof-of-concept superiority trial, directly compared two dose levels of once-weekly fosravuconazole with the current standard of care, daily itraconazole, both in combination with surgery at 6 months after treatment onset [6]. The enrolment and treatment of 104 patients in the trial under the challenging conditions in Sudan was made possible through the dedication of the MRC and the trust they cultivated with affected communities [15,19].

## Paving the path to improved treatment options: A new dawn for mycetoma management

Traditionally, eumycetoma treatment has relied on a combination of antifungal medications and surgery, a strategy often characterised by prolonged treatment duration [3]. Patients usually endure months, sometimes years, of antifungal therapy, placing a significant burden on their lives and livelihoods. The existing antifungal medications, while somewhat effective, often fail to eradicate mycetoma infection, leading to recurrence and the need for further treatment [20]. Many antifungal drugs come with a range of side effects, some potentially severe, impacting patient adherence and overall well-being. Furthermore, many antifungals are teratogenic, necessitating imposing contraception on the patient for a long time due to the long treatment period. This may impact the family, particularly because mycetoma affects mostly relatively young generation [1,21–24]. Surgery, while often necessary to remove infected tissue, can be disfiguring and result in loss of function, further adding to the patient’s physical and emotional suffering [25–27].

The trial demonstrated the potential for using fewer pills once per week compared to the twice-daily pills of the current standard (2 vs. 28 capsules per week). This translates to a reduced treatment burden and an expected improvement in patient adherence. Fosravuconazole, with its favourable safety profile, carries less risk of drug–drug interaction, does not have to be taken with food/drink and, with a lower pill burden, provides an alternative for safe and effective treatment of patients with eumycetoma.

## Reaping the rewards: Advantages that fuelled success

The world’s first-ever randomised, double-blind clinical trial for a new mycetoma treatment was made possible because of several key factors. The first is a genuine trust between the MRC and the communities it serves. This bond, carefully nurtured over years of providing compassionate and high-quality care for mycetoma patients, proved invaluable during the trial. The enrolment of 104 patients, a significant number for a disease often stigmatised and misunderstood, speaks volumes about the community’s faith in the MRC. People felt confident entrusting their health to a familiar institution with a proven track record of care. The MRC’s good patient management and follow-up care were instrumental in identifying potential participants from the high-risk mycetoma areas in Sudan [15,19].

Training programmes were an essential part of conducting the trial. These programmes covered a wide range of essential areas that would ensure accurate data collection, analysis, and reporting while adhering to the highest international standards. The skills developed included Good Clinical Practice, which familiarised the team with ethical and scientific quality standards for designing, conducting, recording, and reporting trials involving human subjects; Good Clinical Laboratory Practice, which ensured the reliability, quality, and integrity of data generated for by standardising laboratory procedures; and Good Financial Practice, which ensured transparent and accountable financial management of the trial while adhering to ethical and regulatory requirements. Developing these skills also instilled a deep understanding of ethical principles, informed consent, patient confidentiality, and research integrity. The MRC developed effective strategies for identifying potential participants, addressing their concerns, and ensuring their continued participation throughout the trial. The trial introduced and refined the use of cutting-edge diagnostic tools to enhance disease diagnosis, monitoring, and treatment outcomes [28]. This investment in capacity building has had a profound and lasting impact on the MRC. The centre is now equipped with a highly skilled workforce capable of conducting high-quality research that meets international standards.

Furthermore, the MRC’s infrastructure and equipment have significantly improved, enhancing both research capabilities and patient care. Upgraded laboratories facilitate advanced diagnostics and more rigorous clinical trials, while state-of-the-art medical equipment streamlines treatment processes. These enhancements not only improve the quality of care for patients but also position the MRC as a leading centre for mycetoma research, attracting more collaboration and funding opportunities. Ultimately, these advancements support the MRC’s mission of providing equitable healthcare access and better outcomes for affected populations (www.mycetoma.edu.sd).

DNDi honoured the MRC with the Project of the Year Award for 2023. This award recognised the MRC’s ability to maintain high research standards despite challenging conditions. The accolade not only celebrates the MRC’s achievement but also underscores the importance of quality clinical trials in challenging environments, particularly for diseases affecting vulnerable populations.

The trial findings provide valuable insights into the efficacy and safety profiles of new treatment regimens for mycetoma, highlighting their potential as viable alternatives. However, they also demonstrate that itraconazole remains an effective option when patients adhere to the prescribed regimen and when it is accessible. This emphasises the critical role of patient compliance and medication availability in achieving optimal treatment outcomes. Overall, the results inform clinical practice and underscore the need for strategies that enhance adherence and ensure effective treatments are accessible to those in need.

## Navigating uncharted territory: Challenges encountered

The main challenges of conducting a randomised, double-blind clinical trial include keeping the blinding intact, high costs and complexity, recruiting and maintaining participants, avoiding bias in randomisation, ensuring participants stick to treatment plans, and strict eligibility criteria (requirement to fulfil all study criteria) to reduce risks to the patients.

These challenges can affect the accuracy and reliability of the study results. The MRC overcame these challenges through meticulous patient selection using the protocol eligibility criteria, close follow-up of patients, especially during the COVID-19 pandemic, and ensuring timely medication dispensing. Patient safety was regularly assessed during scheduled and unscheduled study visits: physical examinations to determine the patient’s vital signs and review the mycetoma lesion; laboratory examinations to assess various body functions, including renal, adrenal, hepatic, and cardiovascular systems, immune profiling, and imaging studies, such as conventional X-rays, ultrasounds, and MRIs. Adverse events that were related or not related to the investigational product were also routinely assessed, documented, and reported to the sponsor and national ethics and regulatory committees. Drug serum levels were evaluated in collaboration with Radboud University Medical Center, Nijmegen, Netherlands, and analysed by Uppsala University, Sweden. Identification of causative organisms down to the species level, including analysis of their profiles and genetic backgrounds, was done in collaboration with Erasmus University Medical Center, Rotterdam, Netherlands.

Mycetoma, often relegated to the periphery of global health priorities, suffers from a chronic lack of research funding and attention [1,29]. The resulting knowledge gap cast a long shadow over the trial, manifesting in several critical areas. A limited, accurate understanding of the epidemiological characteristics of mycetoma in Sudan, including its prevalence, geographic distribution, and risk factors, made it difficult to design effective patient recruitment strategies and generalise findings to the wider population. Furthermore, as this was the inaugural clinical trial, the study team needed to establish clear definitions for study endpoints, criteria for determining a cure, and imaging procedures to assess disease progression, among other tasks. Consequently, a randomised, double-blind, phase 2, proof-of-concept superiority trial design was chosen. Today, armed with the data and insights gained from this study, we are in a stronger position to design additional trials focussed on mycetoma.

The trial encountered significant challenges due to the unique socioeconomic context and varying health literacy levels of the target population [30,31]. Many potential participants were unfamiliar with the concept of clinical trials, leading to fears and misconceptions often stemming from historical exploitation in medical research, particularly within marginalised communities [28]. Additionally, Sudan vast geography and limited transportation access to the MRC for follow-up visits created substantial barriers to participation and adherence [17,25]. Many patients, already facing financial difficulties due to their illness, had to make tough choices between seeking treatment and meeting their basic needs. To address these challenges, the MRC implemented several measures to promote ongoing participation in the study, including increased health education sessions and awareness events in endemic regions.

In low-resource settings, effectively communicating complex medical information can be difficult due to patients’ limited health education and socioeconomic challenges. To mitigate this, MRC staff received extensive training in communication skills to ensure obtaining accurate informed consent, emphasise the importance of treatment adherence and follow-up visits to patients, and encourage them to report any abnormal symptoms or side effects. All study participants underwent a thorough informed consent process. Illiterate patients had an independent witness to assist them with the consent form before it was signed. Informed assent was also provided for patients under 18 years of age. The informed consent and assent forms were made available in both English and Arabic to ensure local relevance. Additionally, illustrations of the study procedures were created and shared with patients to enhance their understanding and adherence to medications and follow-up.

The trial timeline coincided with periods of significant external turmoil, which compounded the challenges faced by the research team and patients. The emergence of the COVID-19 pandemic introduced unprecedented difficulties, marked by lockdowns and travel restrictions that severely limited patient access to the MRC. These disruptions in healthcare services affected patient recruitment, follow-up visits, and the overall continuity of the trial. Additionally, ongoing political unrest and instability in Sudan further exacerbated these challenges.

Despite these formidable obstacles, the MRC demonstrated remarkable perseverance and adaptability, ultimately leading to the completion of the trial. The staff enhanced communication with patients through mobile phones and utilised WhatsApp. In some situations, the medications were directly delivered to patients in their villages to avoid dosing time disruption. The insights gained from navigating these challenges provide valuable lessons for future research endeavours in resource-limited settings, emphasising the need for community engagement, culturally sensitive approaches, and robust logistical planning to overcome systemic barriers and improve health outcomes for all [28].

Securing funding for mycetoma research and clinical trials proved challenging as it often falls outside the priority lists of many funding organisations. Furthermore, the true extent of the disease remains unclear, complicating efforts to persuade potential funders to offer support. The preference of some funders for mycetoma basic epidemiological studies over clinical trials of treatments has added another layer of difficulty to funding efforts. In response, DNDi and the MRC successfully and actively sought full funding for the study through continuous advocacy and awareness initiatives. The inclusion of mycetoma in the WHO’s list of neglected tropical diseases helped raise its profile. Many funders, including GHIT, generously supported the trial, contributing to its advancement.

## Charting a course for the future: Lessons learned and recommendations

This pioneering clinical trial for mycetoma treatment is a significant milestone and a cause for optimism, demonstrating the value of persistence in overcoming challenges. The clinical trial has shed light on three critical areas that require considerable enhancement to improve research outcomes in endemic regions. Firstly, the trial’s success relied heavily on robust community engagement, highlighting the necessity of evolving from passive to active community participation. This involves incorporating local perspectives into the research design, cultivating long-term, culturally sensitive relationships to foster trust, and facilitating informed decision-making through transparent, culturally appropriate communication. Secondly, the trial underscored the importance of a robust research infrastructure that goes beyond researchers’ enthusiasm. This includes ensuring sustainable laboratory capacity through consistent access to equipment, reagents, and quality assurance programmes, as well as prioritising human capital development via comprehensive training and professional growth opportunities for local healthcare professionals. Thirdly, the trial revealed the inextricable link between clinical trial success and the broader healthcare landscape, emphasising the need for healthcare system strengthening. This involves tackling systemic healthcare disparities, improving access to basic services and diagnostic facilities, and addressing the shortage of trained professionals to build a more resilient and supportive research environment. By focussing on these interconnected areas, future trials can build upon the fosravuconazole RCT, significantly enhancing the effectiveness and impact of research initiatives in endemic regions. This holistic approach promises to advance medical research in challenging environments, ultimately leading to improved health outcomes for affected populations and paving the way for more successful and impactful healthcare and research endeavours in the future.

In conclusion, this clinical trial stands as a testament to the power of determination, collaboration, and community engagement in overcoming significant challenges. Despite the backdrop of political instability, the COVID-19 pandemic, and remote and hard-to-reach populations, the MRC successfully navigated its way through extraordinary resilience and resourcefulness. Several key advantages, including adequate patient enrolment, appropriate patient management as per protocol, and strong community trust, underpinned the success of the trial. Moreover, the research initiative catalysed substantial capacity building within the MRC, enhancing its research infrastructure and staff expertise. This pioneering effort not only advanced mycetoma treatment but also offers invaluable lessons for conducting clinical research in resource-limited settings [28]. It underscores the importance of investing in neglected disease research, strengthening healthcare infrastructure in endemic regions, and engaging affected communities as partners. The success of this trial paves the way for larger-scale studies and offers hope for patients with mycetoma, providing an alternative treatment for this long-neglected disease [15,19]. Ultimately, this landmark trial demonstrates that with unwavering commitment and innovative approaches, significant strides can be made in addressing global health challenges, even in the most challenging environments.

## References

[1] Zijlstra EE, van de Sande WWJ, Welsh O, Mahgoub ES, Goodfellow M, Fahal AH. Mycetoma: a unique neglected tropical disease. Lancet Infect Dis. 2016;16(1):100–12. doi: 10.1016/S1473-3099(15)00359-X 26738840

[2] Fahal AH. Mycetoma: a thorn in the flesh. Trans R Soc Trop Med Hyg. 2004;98(1):3–11. doi: 10.1016/s0035-9203(03)00009-9 14702833

[3] Elkheir LYM, Haroun R, Mohamed MA, Fahal AH. *Madurella mycetomatis* causing eumycetoma medical treatment: the challenges and prospects. PLoS Negl Trop Dis. 2020;14(8):e0008307. doi: 10.1371/journal.pntd.0008307 32853199 PMC7452721

[4] Scolding P, Fahal A, Yotsu RR. Drug therapy for mycetoma. Cochrane Database Syst Rev. 2018;2018(7):CD013082. doi: 10.1002/14651858.cd013082

[5] Welsh O, Al-Abdely HM, Salinas-Carmona MC, Fahal AH. Mycetoma medical therapy. PLoS Negl Trop Dis. 2014;8(10):e3218. doi: 10.1371/journal.pntd.0003218 25330342 PMC4199551

[6] Fahal AH, Ahmed ES, Bakhiet SM, Bakhiet OE, Fahal LA, Mohamed AA, et al. Two dose levels of once-weekly fosravuconazole versus daily itraconazole in combination with surgery in patients with eumycetoma in Sudan: a randomised, double-blind, phase 2, proof-of-concept superiority trial. Lancet Infect Dis. 2024;24(11):1254–65. doi: 10.1016/S1473-3099(24)00404-3 39098321

[7] Fahal AH, Ahmed KO, Saeed AA, Elkhawad AO, Bakhiet SM. Why the mycetoma patients are still neglected. PLoS Negl Trop Dis. 2022;16(12):e0010945. doi: 10.1371/journal.pntd.0010945 36580447 PMC9799285

[8] Hay RJ, Asiedu KB, Fahal AH. Mycetoma—a long journey out of the shadows. Trans R Soc Trop Med Hyg. 2021;115(4):281–2. doi: 10.1093/trstmh/traa162 33313922

[9] Hay RJ, Fahal AH. Mycetoma: an old and still neglected tropical disease. Trans R Soc Trop Med Hyg. 2015;109(3):169–70. doi: 10.1093/trstmh/trv003 25667234

[10] Fahal AH, Hassan MA. Mycetoma. Br J Surg. 1992;79(11):1138–41. doi: 10.1002/bjs.1800791107 1467883

[11] Abbas M, Scolding PS, Yosif AA, El Rahman RF, El-Amin MO, Elbashir MK, et al. The disabling consequences of mycetoma. PLoS Negl Trop Dis. 2018;12(12):e0007019. doi: 10.1371/journal.pntd.0007019 30532253 PMC6312340

[12] Ahmed AOA, van Leeuwen W, Fahal A, van de Sande W, Verbrugh H, van Belkum A. Mycetoma caused by *Madurella mycetomatis*: a neglected infectious burden. Lancet Infect Dis. 2004;4(9):566–74. doi: 10.1016/S1473-3099(04)01131-4 15336224

[13] Mohamed ESW, Seif El Din N, Fahal AH. Multiple mycetoma lung secondaries from knee eumycetoma: an unusual complication. PLoS Negl Trop Dis. 2016;10(7):e0004735. doi: 10.1371/journal.pntd.0004735 27442512 PMC4956165

[14] Zaid DM, Bakheet OE, Ahmed ES, Abdalati F, Mhmoud NA, Mohamed ESW, et al. Multiple extensive *Madurella mycetomatis* eumycetoma lesions: a case report and review of the literature. Trans R Soc Trop Med Hyg. 2021;115(4):411–4. doi: 10.1093/trstmh/traa164 33406268

[15] Fahal AH, Ahmed IS, Saaed AA, Smith DJ, Alves F, Nyaoke B, et al. Hope amidst neglect: Mycetoma Research Center, University of Khartoum. A holistic management approach to achieve the United Nations’ Sustainable Development Goals. PLoS Negl Trop Dis. 2024;18(9):e0012420. doi: 10.1371/journal.pntd.0012420 39235990 PMC11376558

[16] Elkheir LYM, Delaye P-O, Penichon M, Eadie K, Mohamed MA, Besson P, et al. Emerging therapeutics: the imidazo[1,2-b]pyridazine scaffold as a novel drug candidate for eumycetoma, a neglected tropical disease. Eur J Med Chem. 2024;277:116720. doi: 10.1016/j.ejmech.2024.116720 39142148

[17] Bakhiet SM, Fahal AH, Musa AM, Mohamed ESW, Omer RF, Ahmed ES, et al. A holistic approach to the mycetoma management. PLoS Negl Trop Dis. 2018;12(5):e0006391. doi: 10.1371/journal.pntd.0006391 29746460 PMC5944909

[18] Fahal A, Mahgoub ES, El Hassan AM, Abdel-Rahman ME, Alshambaty Y, Hashim A, et al. A new model for management of mycetoma in the Sudan. PLoS Negl Trop Dis. 2014;8(10):e3271. doi: 10.1371/journal.pntd.0003271 25356640 PMC4214669

[19] Fahal A, Smith DJ, Nyaoke B, Asiedu K, Falves F, Warusavithanas S, et al. Towards enhanced control of mycetoma: a roadmap to achieve the UN’s sustainable development goals by 2030. Trans R Soc Trop Med Hyg. 2024;118(10):646–51. doi: 10.1093/trstmh/trae016 38530874 PMC11907642

[20] Fahal AH, Rahman IA, El-Hassan AM, Rahman MEAEL, Zijlstra EE. The safety and efficacy of itraconazole for the treatment of patients with eumycetoma due to *Madurella mycetomatis*. Trans R Soc Trop Med Hyg. 2011;105(3):127–32. doi: 10.1016/j.trstmh.2010.11.008 21247608

[21] Musa EA, Abdoon IH, Bakhiet SM, Osman B, Abdalla SA, Fahal AH. Mycetoma management and clinical outcomes: the Mycetoma Research Center experience. Trans R Soc Trop Med Hyg. 2023;117(1):12–21. doi: 10.1093/trstmh/trac069 35903002

[22] Hay RJ. Ketoconazole: a reappraisal. Br Med J (Clin Res Ed). 1985;290(6464):260–1. doi: 10.1136/bmj.290.6464.260 3917777 PMC1417545

[23] Mahgoub ES, Gumaa SA. Ketoconazole in the treatment of eumycetoma due to *Madurella mycetomii*. Trans R Soc Trop Med Hyg. 1984;78(3):376–9. doi: 10.1016/0035-9203(84)90126-3 6087513

[24] Venugopal PV, Venugopal TV. Treatment of eumycetoma with ketoconazole. Australas J Dermatol. 1993;34(1):27–9. doi: 10.1111/j.1440-0960.1993.tb00844.x 8240184

[25] Mohamed ESW, Bakhiet SM, El Nour M, Suliman SH, El Amin HM, Fahal AH. Surgery in mycetoma-endemic villages: unique experience. Trans R Soc Trop Med Hyg. 2021;115(4):320–3. doi: 10.1093/trstmh/traa194 33515452

[26] Suleiman SH, Wadaella ES, Fahal AH. The surgical treatment of mycetoma. PLoS Negl Trop Dis. 2016;10(6):e0004690. doi: 10.1371/journal.pntd.0004690 27336736 PMC4919026

[27] Wadal A, Elhassan TA, Zein HA, Abdel-Rahman ME, Fahal AH. Predictors of post-operative mycetoma recurrence using machine-learning algorithms: the Mycetoma Research Center experience. PLoS Negl Trop Dis. 2016;10(10):e0005007. doi: 10.1371/journal.pntd.0005007 27798643 PMC5087941

[28] Omer RF, Ahmed ES, Ali BM, Alhaj HE, Bakhiet SM, Mohamed ESW, et al. The challenges of recruitment in clinical trials in developing countries: the Mycetoma Research Centre experience. Trans R Soc Trop Med Hyg. 2021;115(4):397–405. doi: 10.1093/trstmh/traa165 33484566

[29] van de Sande W, Fahal A, Ahmed SA, Serrano JA, Bonifaz A, Zijlstra E, et al. Closing the mycetoma knowledge gap. Med Mycol. 2018;56(suppl_1):153–64. doi: 10.1093/mmy/myx061 28992217

[30] Hassan R, Deribe K, Simpson H, Bremner S, Elhadi O, Alnour M, et al. Individual risk factors of mycetoma occurrence in eastern Sennar locality, Sennar State, Sudan: a case-control study. Trop Med Infect Dis. 2022;7(8):174. doi: 10.3390/tropicalmed7080174 36006266 PMC9412883

[31] Ahmed AAO, van de Sande WWJ, Fahal A, Bakker-Woudenberg I, Verbrugh H, van Belkum A. Management of mycetoma: major challenge in tropical mycoses with limited international recognition. Curr Opin Infect Dis. 2007;20(2):146–51. doi: 10.1097/QCO.0b013e32803d38fe 17496572

